# Supporting Older People Experiencing Homelessness and Memory Problems in Hostels: Learning From an Ethnographic Study

**DOI:** 10.1093/geront/gnae187

**Published:** 2024-12-18

**Authors:** Penny Rapaport, Gill Livingston, Jill Manthorpe, Caroline Shulman, Garrett Kidd, Ava Mason, Martin Knapp, Sophie Nadia Gaber

**Affiliations:** Division of Psychiatry, University College London, London, UK; North London Mental Health Partnership, NHS Foundation Trust, London, UK; Division of Psychiatry, University College London, London, UK; North London Mental Health Partnership, NHS Foundation Trust, London, UK; NIHR Policy Research Unit in Health and Social Care Workforce, King’s College London, London, UK; NIHR Applied Research Collaboration (ARC) South London, London, UK; Marie Curie Palliative Care Research Department, Division of Psychiatry, University College London, London, UK; Division of Psychiatry, University College London, London, UK; Division of Psychiatry, University College London, London, UK; Care Policy and Evaluation Centre, London School of Economics and Political Science, London, UK; Division of Psychiatry, University College London, London, UK; Department of Women’s and Children’s Health, Healthcare Services and e-Health, Uppsala University, Uppsala, Sweden

**Keywords:** Dementia, Homelessness, Housing and health, Qualitative research methods, Staff support

## Abstract

**Background and Objectives:**

Older people with memory problems living in temporary hostel accommodation have longer stays and higher care needs than those without memory problems. In this ethnographic study, we aimed to elucidate how staff currently support older hostel residents with memory problems, what contextual factors determine support given, and what facilitates positive and meaningful outcomes for staff and residents.

**Research Design and Methods:**

We conducted interviews and participant observations with older people (≥50 years) experiencing memory problems and homelessness (interviews *n* = 17, observations *n* = 13), hostel staff and managers (interviews *n* = 15, observations *n* = 20) from 7 residential facilities (6 hostels and 1 care home), and health and social care practitioners (interviews *n* = 17, observations *n* = 7), from September 2021 to December 2022 in London, England. We analyzed thematically from a critical realist position.

**Results:**

We identified 4 overarching themes: (1) compensatory strategies and routines, (2) hostels are not homes, (3) meeting challenging interactions with compassion, and (4) facilitating opportunities for meaningful interactions. Social interactions for people with memory problems were restricted and, although sheltered by living in hostels, this did not equate to safety or provide opportunities for positive interactions.

**Discussion and Implications:**

Staff worked hard to connect with older residents with memory problems, in resource- and time-poor contexts, often left to provide care beyond their roles in contexts of unmet need. Our ethnographic account has informed co-design of a support intervention for hostel staff working with older people with memory problems, alongside recommendations for policy and practice.

## Background and Objectives

The population experiencing homelessness is aging in the UK and the United States. Approximately 50% of those experiencing homelessness in the United States are aged 50 years and older (Kushel et al., 2023), with the population aged older than 65 years and experiencing homelessness predicted to triple by 2030 (Culhane et al., 2019). The intersection between homelessness and Alzheimer’s disease and related dementias (ADRD) is increasingly recognized, with those experiencing homelessness significantly more likely to have ADRD (Booth et al., 2024), cognitive (Brown et al., 2017; Jutkowitz et al., 2019) and associated functional impairment (Suh et al., 2022) than stably housed populations.

Diagnosis of dementia and identification of cognitive impairments in older people experiencing homelessness are challenging, with multiple causal factors contributing to poorer brain health. People experiencing homelessness have accelerated aging (Brown et al., 2017; Suh et al., 2022) associated with faster cognitive decline and poorer health outcomes relative to stably housed populations (Jutkowitz et al., 2019). This often relates to long-standing and interacting physical and mental health conditions, substance misuse, and brain injury. Although not formally diagnosed, many older people experiencing homelessness present with cognitive and functional impairment like ADRDs. In this study, we use the term “memory problems” to include those exhibiting dementia-type symptoms, often without formal diagnosis.

Understandably, research has focused on prevalence, risk factors, and associations with cognitive impairment and ADRDs in those experiencing homelessness (Booth et al., 2024; Jutkowitz et al., 2019), who face deep, entrenched social exclusion with multiple structural barriers to diagnosis, care, and support (O’Carroll & Wainwright, 2019; Shulman et al., 2018). Therefore, not only are existing prevalence rates likely underestimated, but less is understood about how best to support this population and what service and housing solutions may look like (Humphries & Canham, 2021; Manthorpe et al., 2019). Older people with memory problems experiencing homelessness have high personal and social care needs and utilize more emergency and out-of-hours services than noncognitively impaired age-equivalent populations (Jutkowitz et al., 2019; Manthorpe et al., 2019). It can also be harder for those experiencing homelessness to move on from temporary accommodation due to complex ill health and multimorbidity and lack of suitable, tailored accommodation (Humphries & Canham, 2021; Manthorpe et al., 2019; O’Connor et al., 2023). For the purpose of this study, we are focusing upon homelessness hostel accommodation. Although there is no single-agreed definition of a hostel in the UK, hostels offer basic temporary accommodation and tend to be provided by specialist housing associations, registered social landlords, voluntary organizations, and charities. They are generally commissioned by local authorities to provide short-term accommodation (usually with a 2-year maximum stay, often shorter) with specific entry criteria and costs are mainly covered by welfare housing benefits. Generally, staff will offer support finding settled accommodation. Typically, hostel residents will have private rooms with shared bathroom and cooking facilities, sometimes meals are provided. Hostels differ from night shelters in the UK, which tend to be more restricted offering people a few nights respite from the streets.

Qualitative and ethnographic studies are increasingly conducted to inform policy and practice from the perspective of those with lived experience of homelessness and those supporting them (O’Carroll & Wainwright, 2019; Shulman et al., 2018). Studies have foregrounded the voices of older people experiencing homelessness, focusing on pathways into homelessness, the impact of homelessness, challenges to moving on, and the meaning of aging in the right place (Canham et al., 2023; Om et al., 2022). Studies focusing on those experiencing cognitive impairment highlight the intersectional experiences of disability, illness, socioeconomic disadvantage, and disempowerment (Townsend et al., 2019), and loneliness as a common yet heterogenous experience (Yuan et al., 2024). Absent from these interview-based studies is a relational account and exploration of interactions between those experiencing homelessness and memory problems and those supporting them.

In summary, there are calls for support and housing-based interventions to meet the unmet needs of this aging population (Booth et al., 2024; Jutkowitz et al., 2019; Suh et al., 2022), which foreground the intersection of aging and homelessness in the context of cognitive decline (Weldrick & Canham, 2023). In this qualitative ethnographic study, we aimed to conduct in-depth interviews and observations of interactions between staff and residents in hostel accommodation to elucidate: (i) how staff currently support older hostel residents with memory problems; (ii) what contextual factors determine current support; and (iii) what facilitates positive and meaningful outcomes for staff and residents.

## Research Design and Methods

### Study Design

We conducted a multisite-focused ethnographic study (Knoblauch, 2005) combining in-depth interviews and participant observations in services supporting older people experiencing memory problems and homelessness. We used step-in step-out ethnographic methods, allowing for longer and shorter phases of engagement (O’Reilly, 2012). This is appropriate for well-defined social interactions and time-specific events, especially in health and care settings (Leverton et al., 2021).

### Setting and Participants

#### In-depth interviews

We recruited hostel staff and managers working with older residents in six complex needs hostels and one specialist care home in London (seven facilities in total) run by five charitable providers. We selected services of different sizes and levels of on-site support for individuals varied in age, physical and mental health needs. We initially approached hostel managers to explain the study and attended team meetings to discuss the study with staff, providing information sheets, and then made appointments with staff to record their consent to participation and conduct in-person interviews. We used posters around hostels inviting participation.

We recruited people experiencing homelessness with self-reported memory problems aged 50 or older, living in hostel accommodation or a specialist care facility for those who have experienced homelessness. The rationale for excluding those currently rough sleeping and including those aged 50 and older is detailed elsewhere, but we wished to study in a place where a potential intervention was possible (Rapaport et al., 2023). We decided to exclude those lacking capacity to consent to participate who may struggle to participate in a qualitative interview due to the severity of impairment. We identified potentially eligible hostel residents through discussion with hostel staff who approached these individuals and provided an easy read information sheet. If residents agreed to be approached, a team researcher was introduced to them in person to discuss the study and take consent (see Supplementary Appendix 1 for detail).

We recruited housing, health, and social care practitioners (HSCPs) and managers involved in commissioning or providing support for older people experiencing homelessness and memory problems via existing networks from participating voluntary or charitable organizations and National Health Service (NHS) and local authority (LA) services in London, Sussex, and Yorkshire. We contacted potential participants by email and sent information sheets, arranging a subsequent online meeting if they wished.

We purposively selected participants of differing ages, genders, ethnicities, nationalities, roles, and work experience (professionals), self-reported physical health status, care needs, and substance use (older people). Background and demographics questionnaires were administered prior to conducting interviews.

Between September and December 2021, we interviewed 17 older people living with memory problems, 17 HSCPs, and 15 hostel staff and managers. We recruited hostel staff and older people from six complex needs hostels and one specialist care home (sizes ranged from 29 to 79 beds); three provided accommodation for men only, four were for older people only, and one hostel had an on-site care service (see Table 1).

**Table 1.** Demographic Characteristics of Interview Participants

		People with memory problems	Hostel staff and managers	Health and social care practitioners
Characteristic	Category	*n* (%) or mean (*SD*)	*n* (%) or mean (*SD*)	*n* (%) or mean (*SD*)
*n*		17	15	17
Age		62.3 (8.7)	42.2 (10.4)	42.2 (10.4)
Gender	Female	1 (5.8)	10 (66.7)	10 (58.8)
	Male	16 (94.2)	5 (33.3)	7 (41.2)
Ethnicity	White British	11 (64.8)	11 (73.3)	15 (88.2)
	White Irish/Welsh	3 (17.6)	0 (0.0)	0 (0.0)
	White other	0 (0.0)	1 (6.7%)	1 (5.9)
	Asian British	0 (0.0)	2 (13.3)	0 (0.0)
	Asian other	0 (0.0)	0 (0.0)	1 (5.9)
	Black British	2 (11.8)	1 (6.7)	0 (0.0)
	Black other	1 (5.8)	0 (0.0)	0 (0.0)

*Note*: SD = standard deviation.

#### Participant observations

We completed interviews prior to initiating observations to facilitate engagement and build relationships with services. We then purposively approached two hostels and the one specialist care facility that had participated in the interview study. Managers in all three services gave written informed consent for service participation.

Inclusion criteria were identical to the interview study, except we excluded staff leaving work in the next 3 months or older participants moving within the next month. Though we had ethical permission to recruit people living with memory problems lacking capacity to consent, none of the participants approached to participate lacked this capacity. After the managers consented to their facility participating, researchers followed the same processes for identifying, approaching, and consenting residents and staff as for the interviews (see Supplementary Appendix 1).

Between April 2022 and December 2022, we conducted participant observations with 13 older men with memory problems in two complex needs hostels and the one specialist care home (see Tables 2–4). Twenty hostel and care home staff members and seven external HSCPs took part in the participant observations whilst interacting with participating residents.

**Table 2.** Characteristics of Observed Accommodation and Type of Observation

ID Number	1	2	3
Hostel/ home type	Complex needs hostel	Complex needs hostel	Registered care home
*N* observed residents	5	5	3
*N* types of observations^a^			
Interaction in communal area	6	5	12
Care delivery in bedroom	—	3	—
Alone/with friend in bedroom	2	7	8
Keyworker support	3	9	1
Mealtime	—	1	7
Group social activity	1	—	2
On-site nurse/therapy review	6	2	—
Medication administration	—	—	3
Off-site medical appointment	—	2	—
Staff team discussion	7	2	4
Off-site outing	1	1	1
*N* of beds	79	42	29
*N* age ≥ 50 (% memory problems)	50 (20%)	28 (39%)	26 (75%)
Gender (min age)	Men only (35)	Men and women (40)	Men only (50)
Meals (cooking facilities Y/N)	3 meals provided (Y)	3 meals provided (N)	3 meals provided (N)
Maximum stay	2 years	2 years	No
Support for residents	Keyworking Attending appointments Move on planning	Keyworking Attending appointments Move on planning	Keyworking Medication Personal care Laundry Attending appointments
Activities on site	Art therapy Occupational training	None provided	Social groups Watching football Art therapy Yoga

*Notes*: N = no; Y = yes.

^a^Numbers of types of observed interactions exceeds total numbers of interactions reported in article, as certain observations included multiple types of interactions.

**Table 3.** Characteristics of Observation Participants With Memory Problems

Pseudonym	Setting	Age	Ethnicity	Time in current hostel	Seen doctor about memory	Current alcohol use	Physical health needs	Mental health needs (diagnosis)	Support with appointments or finances	Support with personal care or bathing	Support with cooking, laundry or shopping
Ben	Hostel 1	72	White Irish	2 y	N	6–7 times/week	Y	N	Y	N	Y
Sean	Hostel 1	61	White Welsh	1 y 8 m	Y	6–7 times/week	Y	Y (PTSD)	Y	N	N
Simon	Hostel 1	60	White British	15 y	Y	None	Y	N	Y	N	Y
Peter	Hostel 1	64	White British	17 y	N	None	Y	Y (Depression)	Y	N	Y
Charles	Hostel 1	63	White British	1 y 6 m	N	6–7 times/week	N	Y (Not stated)	N	N	Y
Lloyd	Hostel 2	62	Irish Jamaican	8 y 7 m	N	6–7 times/week	Y	Y (Depression/anxiety)	Y	N	Y
Jerome	Hostel 2	78	White British	14 y 9 m	Y	6–7 times/week	Y	Y (Not stated)	N	Y	Y
Lucas	Hostel 2	75	White British	8 y 5 m	Unsure	6–7 times/week	Y	Unsure	Y	Y	Y
Tom	Hostel 2	57	White British	1 y 4 m	N	6–7 times/week	N	Y (Not stated)	N	Y	N
Kenneth	Hostel 2	50	White British	7 m	N	6–7 times/week	Y	Y (Depression /anxiety /OCD)	N	N	Y
Jeff	Care home 3	53	White British	8 y 10 m	Y	6–7 times/week	Y	Y (Depression/paranoia)	Y	N	Y
Wesley	Care home 3	61	Black Caribbean	9 y 3 m	Y	None	Y	N	Y	N	Y
Theo	Care home 3	50	White Irish	4 m	Y	6–7 times/week	Y	Y (Depression/anxiety)	Y	Y	Y

*Notes*: ; N = no ; OCD = obsessive compulsive disorder; PTSD = post-traumatic stress disorder; Y = yes.

**Table 4.** Characteristics of Hostel Staff and Managers in Observations

Characteristic	Category	*n* (%) or mean (*SD*)
Age		42.5 (14.08)
Gender	Female	13 (65)
	Male	6 (30)
	Other	1 (5)
Ethnicity	White British	11 (55)
	White other	3 (15)
	Black African	3 (15)
	Black British	2 (10)
	Asian Indian	1 (5)
Job title	Managers/deputy managers/team leaders	4 (20)
	Complex needs/health/specialist workers	3 (15)
	Support workers	8 (40)
	Project workers	1 (5)
	Care assistant	4 (20)
Time working in current role	<1 year	6 (30)
	1–3 years	6 (30)
	3–5 years	4 (20)
	5–10 years	2 (10)
	10+ years	1 (5)
	Did not say	1 (5)
Time working in homelessness sector	<1 year	5 (25)
	1–3 years	4 (20)
	3–5 years	4 (20)
	5–10 years	2 (10)
	10+ years	4 (20)
	Did not say	1 (5)
Experience working with memory problems	Yes	6 (30)
	No	13 (65)
	Did not specify	1 (5)

*Note*: SD = standard deviation.

### Data Collection

#### In-depth interviews

**Figure 1.**
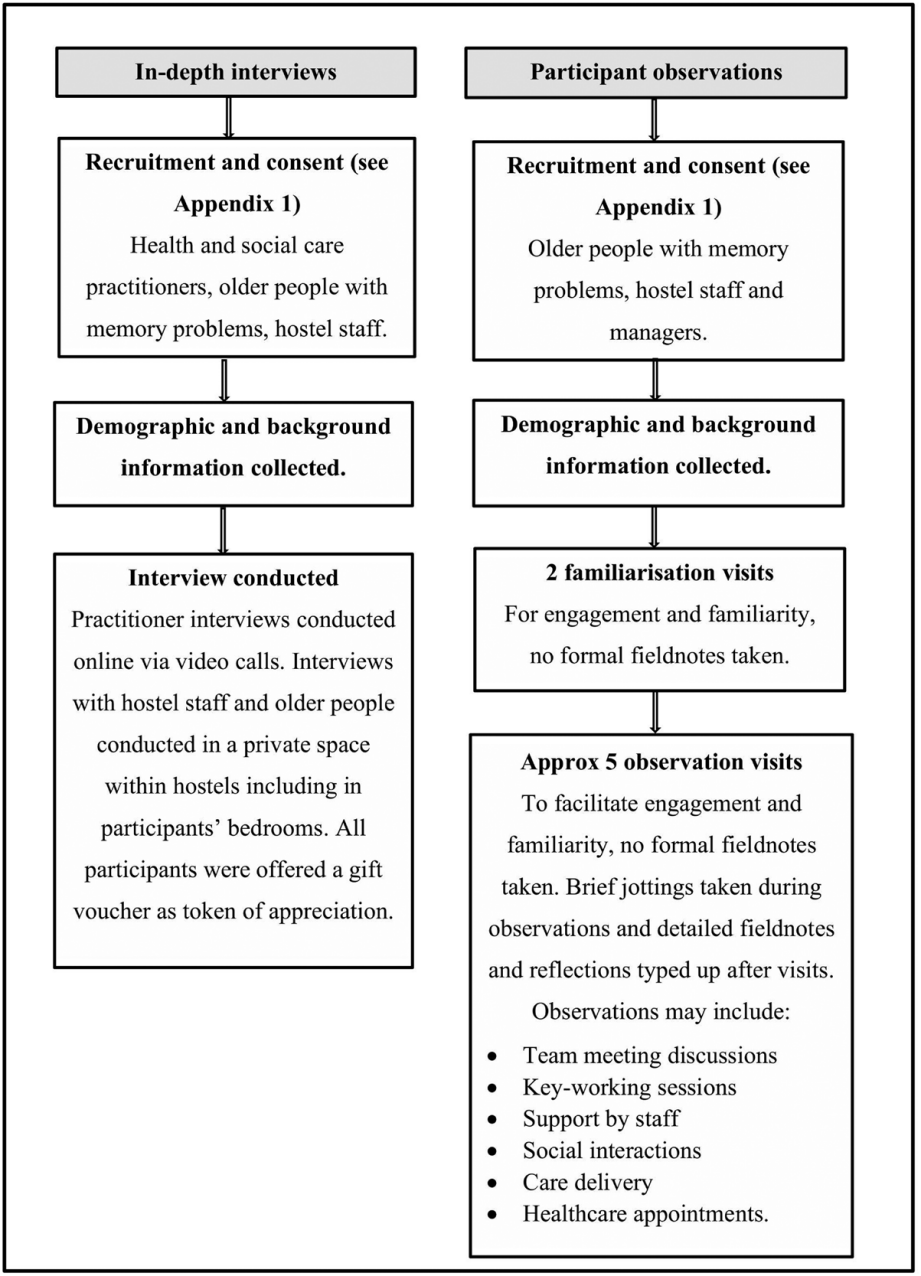
Data collection procedures.

P. Rapaport and G. Kidd conducted all interviews. HSCP interviews were conducted online via video calls. All interviews with hostel staff and residents were conducted in a private space within hostels. We used a semistructured interview schedule based on the literature and research team expertise, with different versions for older people, hostel staff and managers, and HSCPs. Interviews were audio recorded on encrypted digital voice recorders, entered into NVivo 12 software, transcribed verbatim and anonymized. P. Rapaport and G. Kidd met weekly to review recruitment and reflect on initial themes. We ceased interviews after reaching thematic sufficiency, when no further themes emerged from additional interviews (see Figure 1 and Supplementary Appendix 2 for interview schedules).

#### Participant observations

P. Rapaport, G. Kidd, and A. Mason conducted all observations. They conducted up to two visits for each older resident, building a relatively informal rapport without writing fieldnotes. This familiarization period helped build trust, normalized the researchers’ presence, and increased understanding of hostels’ routines (Leverton et al., 2021).

Researchers then conducted approximately five formal observation visits for each participating older person. Observations included interactions with staff and other residents, both in communal areas and in residents’ rooms (checked at each observation and if asked to leave researchers would do so) and included observation of care and support provision and appointments with external providers if agreed (see Figure 1). Observations took place throughout the day to maximize the range of interactions. During observations, researchers took brief notes using a semistructured fieldnote guide (see Supplementary Appendix 2) to ensure consistency between researchers and alignment with our wider research aims. These were typed up and anonymized as soon as possible after the visits. Additionally, during observations, researchers conversed naturalistically using an ethno-interviewing technique to enrich fieldnotes (DeWalt & DeWalt, 2011).

We conducted 60 participant observations over 36.2 hr (median length of observation 17.5 min, range 5–210 min). Observations included welfare checks by staff, discussions between older people and their key workers, visits by care workers, communal mealtimes, group-based activities, medical and other health checks, hospital and future accommodation visits, and team discussions (see Table 2 for details).

### Data Analysis

We took a reflexive thematic analytic approach (Braun & Clarke, 2019) informed by a critical realist position that acknowledges both the impact of social context upon people’s experiences and their material or lived experiences (Rees & Gatenby, 2014). We adopted this theoretical position to reflect how lived experiences of homelessness are undeniably framed by institutional culture and social context, yet we also wanted to foreground the voices and experiences of individuals, resulting in an explanatory rather than just descriptive analysis of social phenomena (O’Carroll & Wainwright, 2019; Rees & Gatenby, 2014). P. Rapaport, G. Kidd, and S. N. Gaber read all data to enhance familiarity. We began by independently and systematically coding each interview transcript (P. Rapaport, G. Kidd, and A. Mason) and each fieldnote (P. Rapaport, G. Kidd, A. Mason, and S. N. Gaber) into meaningful fragments. Two researchers coded 25% of the transcripts and the team met frequently to discuss initial codes and to resolve any discrepancies (Scales et al., 2011). S. N. Gaber then organized the data into preliminary themes, meeting with P. Rapaport to explore commonalities and differences between data sets and perspectives, revising iteratively between interview and observational data, “following the thread” to gain a coherent and comprehensive account (Moran-Ellis et al., 2006). P. Rapaport revisited the whole coded data set directly to check the themes against the data and together P. Rapaport and S. N. Gaber agreed to the final themes. To enhance the findings’ credibility and dependability, initial and subsequent iterations were discussed with the wider team (M. Knapp, G. Livingston, J. Manthorpe, and C. Shulman) and individuals with lived experience of homelessness, but not study participants.

### Ethical Considerations

London (Brighton and Sussex) National Research Ethics Service approved the study (reference: 21/LO/0541). Participants gave written or verbally recorded informed consent prior to interviews or participant observations. As hostels are semipublic spaces, nonparticipating staff, residents, and external visitors were often present. We protected their rights and privacy by restricting observations when nonconsented individuals were present distinguishing between “intensive” and “general” observations, with intensive observation attributable to consented individuals and general observation attributable to no-one in particular (HRA Protocol guidance and template for use in qualitative research 2016:5).

### Reflexivity and Positionality

P. Rapaport is a clinical academic psychologist working with older people with complex needs and cognitive impairment, and G. Kidd and A. Mason are nonclinically trained researchers with a background in psychology, together with the rest of the team we brought to the work our own biases and assumptions about what we would find and met weekly during the project to share reflections and interrogate these assumptions. This was especially important during our participant observations, where we faced dilemmas about how much to “participate,” particularly in an environment where we perceived a support gap that our presence may fill. We adopted a position of “peripheral” observer, gaining an insider perspective without directly participating in the observed care or support. This was not always easy to maintain, and differed for each of us. At times I (P. Rapaport) found myself drawn into a more therapeutic position during conversations with staff and residents. For G. Kidd and A. Mason maintaining this observer position was harder when observing outside of the hostel, for example, when G. Kidd accompanied a participant to a hospital appointment. G. Kidd did intervene when it seemed that the resident was going to miss their appointment and reflected on how the observation may have differed if he had not done so.

## Results

### Qualitative Findings

We identified four themes corresponding to our research objectives: (1) compensatory strategies and routines, (2) hostels are not homes, (3) meeting challenging interactions with compassion, and (4) facilitating opportunities for meaningful interactions.

Interview participants are anonymized and referred to by a descriptor of the interviewee category and a number, observation participants have been given a pseudonym and, where pertinent to context, the professional background of staff is mentioned.

#### Theme 1: Compensatory strategies and routines

Staff used routines, practices, and strategies to support residents with memory problems, balancing patience, flexibility, and responsiveness to provide emotional and practical support that compensated for functional difficulties. Within this theme we explore how staff skillfully work with residents to accommodate and mitigate the potential impact of difficulties, weaving in residents’ own strategies and preferences. We highlight how this can result in tensions for staff and residents, especially when there are different perceptions of whether a strategy or routine activity is helping or hindering. At times, strategies are potentially more useful for staff in reducing the likelihood of conflict or reducing demand on their time but can contribute to institutionalization of residents, especially those with long stays.

##### Subtheme 1: Staff help with remembering and understanding

Memory issues were a barrier to residents’ functioning, but staff found creative ways to act as an “external memory” to help residents to remember:

The gentleman I was talking about who forgets when his money’s coming, his keyworker … she’s like his external memory, organizing his time for him. (Allied health professional 1)

Staff simultaneously served as investigators, tracking down information, as well as advocates, supporting residents to understand complex information related to health and finances:

He often comes to tell his keyworker that he had a message that an appointment had been rescheduled, but he can’t say who gave him that message or when it was rescheduled to. His keyworker investigates this. (Hostel manager 1)

##### Subtheme 2: Importance of routines to support memory

Staff emphasized the importance of personalized routines, developed and negotiated in dialogue between staff and residents. These routines were useful for residents in supporting their memory difficulties through repetition or familiarity, and also aided staff by reducing the likelihood of conflict or disagreement. In the following example, the same strategy is described from the perspective of both a support worker and an older resident. Jeff was a regular drinker and tended to lose track of his alcohol intake and could become aggressive if he felt staff were withholding alcohol:

I noticed on the window ledge a laminated sheet with the schedule of Jeff’s alcohol allocation. He explained that he finds this very helpful as it reminds him what he has had. (Fieldnote)

So, when they say, “but I didn’t have that” we say, “There’s the receipt and there’s the signature.” So, we have measures in place for when they do forget. (support worker 1)

Routines frequently revolved around medication, alcohol, and cigarettes, which provided opportunities for social exchanges between residents and staff, and among residents who visited each other’s rooms. We observed a friendship between two older residents, Lloyd and Tom, in which Lloyd supported Tom’s deteriorating physical health and higher care needs by shopping for him and, in return, Tom supported Lloyd with his memory problems. In the following fieldnote, Lloyd was present when a nurse visited Tom, speaking to the nurse on his friend’s behalf:

Tom looked at Lloyd and told him that his wrist was sore. Lloyd looked at him and said, “tell her,” referring to the nurse who had just left the room. When the nurse returned, Lloyd looked at her and said, “it’s hurting him lady.” (Fieldnote)

Some residents had become so used to rigid routines that they seemed institutionalized, struggling to function without this structure. Wesley, who had lived in the specialist care home for over nine years, had a routine of waking up at the same time each day, eating breakfast, taking his medication, and interacting minimally throughout the day, becoming agitated if people sat at his preferred table. Hostel residents felt that rigidity, together with longer than anticipated stays added to a sense of becoming institutionalized:

Even on the leaflet that you get when you come here, it’s a hostel for three to six months. And then I ask, why the hell am I here, after one and a half, well, one year, four months and I’m still here… And you get institutionalized is the word to use when you come here. (Older resident 1)

##### Subtheme 3: Balancing independence, safety, and risk of isolation

Staff faced dilemmas about keeping residents safe, reducing interactions, and separating them from other residents. Residents with memory problems, especially those with a history of paranoia and physical aggression such as Jeff, were observed isolating themselves as a self-imposed way of minimizing challenging interactions and avoiding “breaking any rules”:

Jeff was clear that he was content in his room and that he stays there to avoid interactions which may trigger aggression. This seemed effective for him although he does live in self-enforced isolation. (Fieldnote)

Although some residents favored time alone in their rooms, staff observed residents engaging in solitary activities for prolonged periods or doing nothing in their rooms. Jerome, who was physically frail and had high care needs was observed to be repeatedly lying in bed copying text from newspaper articles to pass the time:

Choosing their favorite newspaper story and copying out word for word. That doesn’t look like fun. That’s more like an obsessive behavior, but it’s almost like there’s nothing else to do except for drink and do that. (Support worker 2)

Often keeping vulnerable residents safe came at the expense of social interaction. Simon was relocated to a higher floor by staff concerned by his vulnerability, after his TV was stolen, resulting in him becoming socially isolated. Staff were concerned about another resident, Ben, who was isolated on a high floor, feeding the birds in the trees outside his window and referring to them fondly as his “pets”:

Janet said that she was the only person Ben sees, which is why they want to bring him to the ground floor. Janet says Ben refuses to move out of his room because he likes being on the 5th floor to feed his pets. (Fieldnote)

#### Theme 2: Hostels are not a home

Inadequate physical environments and structural constraints limited residents’ support. In hostels, staff were not commissioned to provide care to residents. This resulted in residents’ needs remaining unmet despite staff working beyond commissioned roles and bypassing or accommodating structural constraints. Observed interactions and interviews were often characterized by a sense of absence, either of space for interaction or by staff and residents making do with their surroundings. Despite often long-term residency in hostels, there was an atmosphere of impermanence and tacit acknowledgment in the interviews that there was a lack of suitable homelike accommodation for those experiencing memory problems to move on to. This was less evident in the care home observed where care and support were available, and the accommodation was not temporary.

##### Subtheme 1: Mismatch between resident and environment.—

In private rooms and common areas, there was an absence of “homelike” features, including minimal seating. When visiting residents’ rooms, people would sit on the bed, perch on assistive equipment and mobility aids, or squat in a corner, limiting interactions. When observing care provided by external agencies, care workers seemed ill-equipped to deliver care in the hostel setting or to know how best to respond to residents’ complex presentations. Common spaces appeared neglected and unused due to stark furnishings, without seating or a TV:

The only thing in the common area near Tom’s room was a fire extinguisher. The floor was yellow with marks from the furniture that used to be there. There was a TV on the wall which was never on. The whole common area in this wing of the hostel was unused space. (Fieldnote)

Lack of opportunity or comfortable places for interaction resulted in residents “hanging around” reception areas:

Simon and I were standing up during the entire interaction. There were no chairs in the reception area. He is often there just leaning against the wall saying hello to people. (Fieldnote)

Hostel staff discussed ongoing tension regarding scarcity of suitable onward accommodation, which exacerbated lack of clarity about whether hostels were to function as temporary accommodation, homes, or indeed care homes. Greater alignment between residents and their environment was observed in the specialist care home both during mealtimes, which were provided in a dining room, and group activities, such as decorating a Christmas tree. These activities contributed to a more “homelike” environment, and enabled residents with different memory and physical impairments, like Theo who had a stroke during the observation period and poor mobility, to contribute:

Theo said, “We are doing the Christmas decorations…we did that one as well.” Theo was slowly putting the balls on the tree with one arm only due to his stroke, dropping one on the floor. Theo yawns and takes another ball from the bag which he drops on the floor. (Fieldnote)

##### Subtheme 2: Staff working beyond their roles

Staff worked hard to connect with residents in resource- and time-poor contexts, limited by insufficient equipment or specialist training to adequately support residents. This included few memory aids to help orientate residents:

And how many of those clients will have a clock, a calendar, a diary, or a watch? They generally don’t exist. (Allied health professional 2)

Staff adapted by providing support that extended beyond their roles. We observed a staff member physically lifting Jerome, a wheelchair user, into a car due to a lack of accessible transport, and another staying late to ensure a resident was able to sign a tenancy agreement for new accommodation:

I witnessed Gemma struggle to lift Jerome from his wheelchair into the back seat of the car, without any significant help. (Fieldnote)

Interactions between staff and residents revolved around practical discussions about finances, health, or the state of residents’ rooms rather than opportunities to connect with residents and chat, with staff putting themselves in difficult situations because they would not want to leave a resident struggling:

There is day-to-day care which I just think it’s just completely outside of the staff remit…Even with the residents who have carers, they can’t not do that because, they just wouldn’t, you know they wouldn’t not clean up if somebody had had an accident or their room was in a terrible, terrible state. (Physician 1)

#### Theme 3: Meeting challenging interactions with compassion

Hostel staff demonstrated compassion to support residents in complex and challenging situations. The pervasive social isolation meant that relationships with staff were centrally important to residents with memory problems. Although staff did not necessarily spend extended periods with residents, they worked hard to ensure that they were minimizing any sense of embarrassment or shame that residents may have felt about their impairment and related difficulties.

##### Subtheme 1: Diffusing, distracting, and reframing

Staff would distract residents with memory problems to diffuse tense situations that could erupt into conflicts or aggression. Wesley, although a long-term resident, had severe memory impairment and felt he had only just moved to the hostel:

Wesley shouted, “I shouldn’t be here, I’m waiting for my flat.” Henry, a senior staff member came out of the office and said to Wesley, “Are you here for your fags, here you go.” Henry walked Wesley to the lift and chatted asking “what did you eat this morning?” (Fieldnote)

As a result of long stays, staff often had close connections with residents, like Jerome who had been in the hostel nearly 15 years. Often staff used humor to help calm residents when supporting them with anxiety-provoking situations, such as managing their finances or communicating with services over the phone:

Gemma dialed the number of the bank and turned the call onto loud-speaker so Jerome could hear it. Gemma then almost dropped the statement and Jerome managed to stop it falling. They both laughed. Once the call was answered, it was an automated service. Gemma said, “talking to a robot” and we all laughed. (Fieldnote)

Activities, inside and outside of hostels, provided opportunities for positive reframing, diversions, humor, and distractions from everyday routines. On a day trip to visit a football club, a staff member, Henry, shared a humorous exchange with Theo, who had challenges keeping up with other residents and staff due to mobility problems:

We were in the shop and Henry said, “did you bring your money to pay?” [In relation to the free scarf and badge] …Theo asked Henry “who do you like?” and he said “Rangers, Everton remember…you can smell that grass can’t you?” and Theo looked back and smiled. (Fieldnote)

##### Subtheme 2: Staff sensitively support residents’ health

Hostel staff would piece together fragmented information and fill in gaps for residents, but this was not always possible. Staff compared this process to doing a jigsaw:

I’m going at it from the perspective of maybe this is a piece of the jigsaw, because, you know, unless you work with [someone] for a long- time I don’t think you get the true picture. (Inclusion health nurse 1)

Residents with memory problems expressed heightened anxiety regarding their health, related to understanding and retaining information about their diagnoses or treatments, and attending appointments. Receiving health-related information via emails or texts exacerbated anxiety among residents with memory problems. Staff helped to alleviate health-related anxieties by clarifying and reassuring, by offering to accompany residents to healthcare appointments, as well as visiting residents in hospital. A staff member visited Lucas in hospital and helped to get information about his condition and discharge plans:

Lucas quickly pointed to the nurses’ station, getting distressed saying, “they don’t tell me anything.” Paige said that she could go to the nurses to see what was happening. Paige told Lucas that she had talked with the homelessness team the day before and that they would “come today.” (Fieldnote)

When residents attended healthcare appointments, staff were concerned whether residents understood their health issues or whether they would attend follow-up appointments. Residents faced several challenges when staff were not present to fill in the gaps. Kenneth, who was a regular drinker, was observed struggling to attend a neurology appointment unaccompanied:

Kenneth smiled and said that he knew how to get to the hospital. He suggested that we leave at 12:50 pm, which was not enough time…Kenneth was confused about the location and thought the appointment was for a liver, not a neurology appointment… When we approached the steps of the hospital entrance, Kenneth used the handrail to pull himself up the stairs…Once inside, Kenneth said he had forgotten his letter. (Fieldnote)

#### Theme 4: Facilitating opportunities for meaningful interactions

Staff and other residents provided opportunities for residents to engage in activities that residents perceived as meaningful, and to facilitate social connections inside and outside the hostels, also acknowledging that, although perceived as positive, this was not always possible:

Maximizing independence and maximizing, you know, them doing meaningful things with their life, not being so isolated, initially through kind of professional contacts, but, hopefully through community stuff and not so much dependent on professional staff and maybe even peer groups. (Psychiatrist 1)

Although staff had often got to know residents well, facilitating meaningful interactions was also made harder by the intersecting challenges of homelessness and cognitive impairment as staff were not always aware of residents’ current and past relationships, and, as noted above, often wanted to protect these residents from potential harm.

##### Subtheme 1: Challenges of maintaining relationships and friendships

Narratives of loss and breakdown of relationships pervaded both interviews and observations. Residents became frustrated about not remembering a bereavement or expressed disappointment about strained relations with family. Staff helped by talking with residents about concerns and suggesting ways that residents could (re)connect with family members:

“I haven’t seen my children for some time, what have I done to upset my children?” Susan was understanding but gently challenged why Jeff does not come and call his children but waits for them to contact him. Jeff commented that he does not want to talk by phone he wants to see them and hold them. (Fieldnote)

Many residents lacked a consistent, stable social network or family who might typically support people with memory problems not experiencing homelessness:

It’s not just about our clients, it’s about everything that surrounds them. When we have people with memory problems, it’s like, you know, where are their family? (Support worker 2)

Staff made considerable efforts to support residents with (re)connecting and maintaining relationships with family and friends while simultaneously considering potential risks and the need to protect residents from other people who may have a detrimental influence:

Handover was carried out by Susan… She said that Theo had got a letter from a friend to go to his old hostel, and that he was very happy about this. She said, “he is there now to see his friend, but I had to remind him before he left to be careful, he was legless when he came back, falling over from being that drunk.” (Fieldnote)

##### Subtheme 2: Seeking social interactions and engagement in meaningful activities

The interviews and observations highlighted how hostel staff adopted a nonjudgmental and flexible stance while encouraging befriending and social interactions with residents:

Candice said to Jeff “we have tea, biscuits, crisps and some newspapers to have a little chat, just a bit of a social get together to catch up but if you just want a cup of tea that’s also fine.” Jeff nodded and walked by the table to get some tea, slowly but quietly serving himself. (Fieldnote)

Staff described insufficient opportunities for social interactions or engagement in meaningful activities to address boredom and keep residents stimulated in a positive, nonaggressive, and healthy way, however, this was not always possible. Simon was often observed in the reception area of the hostel:

Simon was waving his arms around and making threatening comments toward the carer and Anne had to intervene. Once Anne had encouraged Simon to return to his room, Anne commented that it is not good for Simon to be here because he does not have anything to do. She also said that the staff in the hostel are not trained to work with people with memory problems. (Fieldnote)

Despite constraints within the hostels, staff were observed engaging in small, positive interactions with residents. Discussing residents’ interests helped foster more trusting, consistent, and supportive relationships between staff and residents:

Wesley was greeted by Susan brightly and he responded, “Bonjour madam”… they carried on discussing boardgames and Wesley commented: “I used to play Monopoly.” Susan responded, “ah really I love Monopoly, what was your favourite place?” (Fieldnote)

## Discussion and Implications

To our knowledge, this is the first ethnographic exploration of interactions between older people experiencing memory problems and homelessness and those supporting them, focused on temporary “hostels” in England. This article extends our earlier research, which foregrounded the lived experiences of memory problems and how this relates to negative assumptions and stigma experienced at the intersection of aging, homelessness, and cognitive impairment (Rapaport et al., 2023). In this study, we observed few opportunities for safe interactions, especially in hostels, where residents spent most of their time alone in sparsely furnished rooms. Boredom has been previously identified as common and profound for those experiencing homelessness (Marshall et al., 2019) and our participants experienced additional barriers to meaningful engagement, needing support which was not always available, to compensate for their cognitive and functional impairments. In line with existing research, isolation, sometimes self-imposed by older people for self-protection (Yuan et al., 2024), was exacerbated by the context and structure of temporary housing which, despite offering shelter, failed to meet the complex functional, emotional and physical needs of participating residents (Humphries & Canham, 2021).

We included a specialist care facility to enhance opportunities to observe interactions between staff and residents, and to access a broader range of potential interactions to understand what facilitates positive and meaningful support for older people experiencing memory problems and homelessness. People living with dementia can benefit from relational interdependence to support maintenance of a coherent narrative and sense of well-being. For those experiencing memory and other cognitive problems in homelessness, staff worked hard to fill in gaps and supported individuals to make sense of what was happening both practically and emotionally.

Across settings, staff endeavored to connect with older residents with memory problems, despite lack of resources, often providing care beyond their roles. Yet, most older residents had significant and varied care needs, posing dilemmas for frontline staff on how to keep residents safe. This aligns with research in both multiple exclusion homelessness, which highlights the intersection of homelessness and the experience of multiple adverse life events (Fitzpatrick et al., 2013) and dementia care (Leverton et al., 2021) that has highlighted challenges faced by staff who are ethically and personally committed to their clients yet dependent on limited statutory resources and requirements of their roles (Renedo, 2014). Despite effectively functioning as part of the “dementia care workforce,” frontline staff in our study lacked professional recognition, training, and support (Manthorpe et al., 2019). Additionally, two-thirds of participating hostel staff were female, predominantly supporting male residents, and average staff age was 42 years. This reflects the English social care workforce more broadly, where 80% of the workforce are female and the average age is 45 years (Skills for Care, 2023). Although not the focus of the study, there was a tendency for female staff to take or be positioned in a maternal role towards residents. This intersecting age and gendered dynamic may have contributed to female staff working beyond support roles and stepping into unacknowledged care provision, a dynamic previously identified in research exploring the impact of gender and age on the homelessness workforce (Galbraith, 2020).

The interviews and observations underscored that hostels should not be seen as homes but many of the older participants had lived in their current accommodation for several years, building attachments to people and place. Recognizing the limited availability of suitable accommodation options, our participants were inadvertently “aging in place” without the positive outcomes typically associated with this approach (Wiles et al., 2012). Undoubtedly, hostels were conceived of as places of transition and as such notions of “liminal homes” may be a useful lens through which to consider the lived experiences of older hostel residents experiencing memory problems (Leibing et al., 2016). Although we witnessed efforts to make the environments more “homelike,” the hostels were stark and institutional and for those with memory problems their functional and cognitive difficulties combined with a lack of resources resulted in them being “stuck in place.” Our findings emphasize the need for more specialist-supported accommodation for those with complex needs including cognitive impairment, who have experienced homelessness, enabling them to “age in the right place” (Canham et al., 2023), in environments that promote autonomy while adapting to evolving needs, especially for progressive conditions like dementia (Humphries & Canham, 2021).

Our study demonstrates that hostel staff remain ill-equipped and unsupported in this work. We have used this study’s findings to co-design, an intervention for staff supporting older hostel residents with memory problems. The intervention, currently undergoing testing in a nonrandomized feasibility trial (https://doi.org/10.1186/ISRCTN28374317), provides psychoeducation on the multiple causes and impact of memory problems, communication strategies, managing distressed behaviors, promoting meaningful interaction and minimizing harm, understanding mental capacity, collaborating with external agencies, and ways for staff to look after themselves and each other. There is a focus throughout on ways to facilitate meaningful positive outcomes for residents and what these may look like. The intervention is delivered face-to-face in small group training sessions, focusing on implementation, and co-facilitated by trained hostel staff member to embed and sustain learning. Since there are complex structural barriers, including at the intersection of agism and homelessness (Weldrick & Canham, 2023), which lead to a lack of suitable accommodation for those experiencing memory problems and homelessness and restricted access to adequate services, staff training alone will not be the solution and we will use the findings from this and the ongoing trial evaluation to influence policymakers.

## Strengths and Limitations

Focused ethnography involves brief but intensive fieldwork, emphasizing collaborative data collection and analytic scrutiny (Knoblauch, 2005). By adopting a team-based ethnographic approach, we explored diverse relational perspectives and social dimensions, enhancing the depth and rigor of our study compared to interview-only approaches (Scales et al., 2011). As the study began during the coronavirus disease 2019 (COVID-19) pandemic, we adapted our processes to accommodate contemporaneous restrictions, and delayed participant observations to fully access the hostels and collect all observational data in person. It may be that the lack of opportunity for interaction and sparse environments were in part a reflection of the COVID-19 restrictions. When visiting hostels, staff and residents told us that since the pandemic there had been a reduction in activities, and also that some residents who had in the past been active and gone outside regularly had not restarted this when restrictions were lifted as their cognitive impairment and frailty made it more difficult for them to regain functioning. Despite data collection challenges, reflecting on our respective backgrounds (personal and professional) and integrating reflections into our fieldnotes helped ameliorate difficulties (Scales et al., 2011). Most older participants with memory problems were White men and as such we have not been able to consider intersectionality of age, gender, and race for people experiencing homelessness and memory problems. Our population reflects the demographics of UK-based hostels (Manthorpe et al., 2019). It differs from the older population experiencing homelessness in other countries, potentially limiting transferability of findings (Brown et al., 2017; Kushel et al., 2023). Additionally, there is a gap in understanding the lived experiences of older women experiencing homelessness in England, especially those from minoritized and migrant communities, which we are beginning to address in our longitudinal, narrative study presented in this special edition (Gaber et al., in press).

## Supplementary Material

gnae187_suppl_Supplementary_Material

## Data Availability

The qualitative data used and analyzed during the current study are available from the corresponding author in fully anonymized form on reasonable request. The study was not preregistered.

## References

[CIT0001] Booth, R. G., Dasgupta, M., Forchuk, C., & Shariff, S. Z. (2024). Prevalence of dementia among people experiencing homelessness in Ontario, Canada: A population-based comparative analysis. Lancet Public Health, 9(4), e240–e249. https://doi.org/10.1016/S2468-2667(24)00022-7 38553143

[CIT0002] Braun, V., & Clarke, V. (2019). Reflecting on reflexive thematic analysis. Qualitative Research in Sport Exercise and Health, 11(4), 589–597. https://doi.org/10.1080/2159676x.2019.1628806

[CIT0003] Brown, R. T., Hemati, K., Riley, E. D., Lee, C. T., Ponath, C., Tieu, L., Guzman, D., & Kushel, M. B. (2017). Geriatric conditions in a population-based sample of older homeless adults. Gerontologist, 57(4), 757–766. https://doi.org/10.1093/geront/gnw011 26920935 PMC5881727

[CIT0004] Canham, S. L., Weldrick, R., Mahmood, A., Patille, R., & Erisman, M. C. (2023). Meanings of aging in the right place for older clients of a temporary housing program. Gerontologist, 64(5), 1–10. https://doi.org/10.1093/geront/gnad151 37930091

[CIT0005] Culhane, D., Treglia, D., Kuhn, R., Doran, K., Byrne, T., & Metraux, S. (2019). The emerging crisis of aged homelessness. http://www.aisp.upenn.edu/aginghomelessness

[CIT0006] DeWalt, K. M., & DeWalt, B. R. (2011). Participant observation: A guide for fieldworkers (Vol. 2). AltaMira Press.

[CIT0007] Fitzpatrick, S., Bramley, G., & Johnsen, S. (2013). Pathways into Multiple Exclusion Homelessness in Seven UK Cities. Urban Studies, 50(1), 148–168. https://doi.org/10.1177/0042098012452329

[CIT0033] Gaber, S., Mattsson, E., Klarare, A., Dawes, J., & Rapaport, P. (In Press). An Intersectional Perspective on Digital health: Longitudinal Narratives and Observations with Older and Middle-Aged Women Experiencing Homelessness. The Gerontologist. 10.1093/geront/gnaf021 PMC11973565 39869439

[CIT0008] Galbraith, J. (2020). Homelessness workers negotiating the relationship between identity and practice: How gender, age and background influence worker-service user relationship. Housing, Theory and Society, 37(2), 198–213. https://doi.org/10.1080/14036096.2019.1584585

[CIT0009] Humphries, J., & Canham, S. L. (2021). Conceptualizing the shelter and housing needs and solutions of homeless older adults. Housing Studies, 36(2), 157–179. https://doi.org/10.1080/02673037.2019.1687854

[CIT0010] Jutkowitz, E., Halladay, C., McGeary, J., O’Toole, T., & Rudolph, J. L. (2019). Homeless veterans in nursing homes: Care for complex medical, substance use, and social needs. Journal of the American Geriatrics Society, 67, 1707–1712. https://doi.org/10.1111/jgs.15993 31206592 PMC6743476

[CIT0011] Knoblauch, H. (2005). Focused ethnography. Forum Qualitative Sozialforschung *Forum: Qualitative Social Research*, 6(3). https://doi.org/10.17169/fqs-6.3.20

[CIT0012] Kushel, M., Moore, T., Birkmeyer, J., Dhatt, Z., Duke, M., Ray Knight, K., & Young Ponder, K. (2023). *Toward a new understanding: The California Statewide Study of people experiencing homelessness*. https://policycommons.net/artifacts/4308721/caspeh_report_62023/5119094/

[CIT0013] Leibing, A., Guberman, N., & Wiles, J. (2016). Liminal homes: Older people, loss of capacities, and the present future of living spaces. Journal of Aging Studies, 37(10), 10–19. https://doi.org/10.1016/j.jaging.2015.12.002 27131274

[CIT0014] Leverton, M., Burton, A., Beresford-Dent, J., Rapaport, P., Manthorpe, J., Azocar, I., Giebel, C., Lord, K., & Cooper, C. (2021). Supporting independence at home for people living with dementia: A qualitative ethnographic study of homecare. Social Psychiatry and Psychiatric Epidemiology, 56(12), 2323–2336. https://doi.org/10.1007/s00127-021-02084-y 33893821 PMC8558284

[CIT0015] Manthorpe, J., Samsi, K., Joly, L., Crane, M., Gage, H., Bowling, A., & Nilforooshan, R. (2019). Service provision for older homeless people with memory problems: A mixed-methods study. Health Services and Delivery Research, 7(9), 1–184. https://doi.org/10.3310/hsdr07090 30830733

[CIT0016] Marshall, C. A., Davidson, L., Li, A., Gewurtz, R., Roy, L., Barbic, S., Kirsh, B., & Lysaght, R. (2019). Boredom and meaningful activity in adults experiencing homelessness: A mixed-methods study. Canadian Journal of Occupational Therapy, 86(5), 357–370. https://doi.org/10.1177/0008417419833402 30987447

[CIT0017] Moran-Ellis, J., Alexander, V. D., Cronin, A., Dickinson, M., Fielding, J., Sleney, J., & Thomas, H. (2006). Triangulation and integration: Processes, claims and implications. Qualitative Research, 6(1), 45–59. https://doi.org/10.1177/1468794106058870

[CIT0018] O’Carroll, A., & Wainwright, D. (2019). Making sense of street chaos: An ethnographic exploration of homeless people’s health service utilization. International Journal for Equity in Health, 18(1), 113. https://doi.org/10.1186/s12939-019-1002-6 31337407 PMC6651952

[CIT0019] O’Connor, C. M. C., Poulos, R. G., Sharma, A., Preti, C., Reynolds, N. L., Rowlands, A. C., Flakelar, K., Raguz, A., Valpiani, P., Faux, S. G., Boyer, M., Close, J. C. T., Gupta, L., & Poulos, C. J. (2023). An Australian aged care home for people subject to homelessness: Health, wellbeing and cost–benefit. BMC Geriatrics, 23(1), 253. https://doi.org/10.1186/s12877-023-03920-3 37106318 PMC10139912

[CIT0020] O’Reilly, K. (2012). Ethnographic methods. Routledge. https://doi.org/10.4324/9780203864722

[CIT0021] Om, P., Whitehead, L., Vafeas, C., & Towell-Barnard, A. (2022). A qualitative systematic review on the experiences of homelessness among older adults. BMC Geriatrics, 22(1), 363. https://doi.org/10.1186/s12877-022-02978-9 35468760 PMC9040287

[CIT0022] Rapaport, P., Kidd, G., Jeraldo, R. E., Mason, A., Knapp, M., Manthorpe, J., Shulman, C., & Livingston, G. (2023). A qualitative exploration of older people’s lived experiences of homelessness and memory problems – stakeholder perspectives. BMC Geriatrics, 23(1), 556. https://doi.org/10.1186/s12877-023-04250-0 37700235 PMC10498566

[CIT0023] Rees, C., & Gatenby, M. (2014). Critical realism and ethnography. In P. K. Edwards, J. O’Mahoney, & S. Vincent (Eds.), Studying organizations using critical realism: A practical guide. (pp. 132–147). Oxford University Press. http://ukcatalogue.oup.com/product/9780199665532.do#.UgtywpLVB8E

[CIT0024] Renedo, A. (2014). Care versus control: The identity dilemmas of UK homelessness professionals working in a contract culture. Journal of Community and Applied Social Psychology, 24(3), 220–233. https://doi.org/10.1002/casp.2162

[CIT0025] Scales, K., Bailey, S., & Lloyd, J. (2011). Separately and together: Reflections on conducting a collaborative team ethnography in dementia care. Enquire, 6, 24–49. http://enquirenottingham.co.uk/images/edition%206%20-%20scales.pdf/

[CIT0026] Shulman, C., Hudson, B. F., Low, J., Hewett, N., Daley, J., Kennedy, P., Davis, S., Brophy, N., Howard, D., Vivat, B., & Stone, P. (2018). End-of-life care for homeless people: A qualitative analysis exploring the challenges to access and provision of palliative care. Palliative Medicine, 32(1), 36–45. https://doi.org/10.1177/0269216317717101 28672115 PMC5758927

[CIT0027] Skills for Care. (2023). *The state of the adult social care sector and workforce in England*. http://www.skillsforcare.org.uk/stateof

[CIT0028] Suh, K., Beck, J., Katzman, W., & Allen, D. D. (2022). Homelessness and rates of physical dysfunctions characteristic of premature geriatric syndromes: Systematic review and meta-analysis. Physiotherapy Theory and Practice, 38(7), 858–867. https://doi.org/10.1080/09593985.2020.1809045 32835565

[CIT0029] Townsend, C., McIntyre, M., Wright, C. J., Lakhani, A., White, P., & Cullen, J. (2019). Exploring the experiences and needs of homeless aboriginal and Torres Strait Islander peoples with neurocognitive disability [Article]. Brain Impairment, 20(2), 180–196. https://doi.org/10.1017/brimp.2019.21

[CIT0030] Weldrick, R., & Canham, S. L. (2023). Intersections of ageism and homelessness among older adults: Implications for policy, practice, and research. Gerontologist, 64(5), 1–7. https://doi.org/10.1093/geront/gnad088 37392069

[CIT0031] Wiles, J. L., Leibing, A., Guberman, N., Reeve, J., & Allen, R. E. (2012). The meaning of “aging in place” to older people. Gerontologist, 52(3), 357–366. https://doi.org/10.1093/geront/gnr098 21983126

[CIT0032] Yuan, Y., Knight, K. R., Weeks, J., King, S., Olsen, P., & Kushel, M. (2024). Loneliness among homeless-experienced older adults with cognitive or functional impairments: Qualitative findings from the HOPE HOME study. BMC Public Health, 24(1), 569. https://doi.org/10.1186/s12889-024-18052-5 38388904 PMC10885402

