# Supplementary material for: Supporting Older People Experiencing Homelessness and Memory Problems in Hostels: Learning From an Ethnographic Study
Source: Gerontologist. 2024 Dec 18;65(4):gnae187. doi: 10.1093/geront/gnae187 (PMC11971685; doi:10.1093/geront/gnae187)
Supplement: gnae187_suppl_Supplementary_Material [file gnae187_suppl_supplementary_material.docx]

# supplementary material

Appendix 1 – Recruitment and consent processes

Appendix 2 – Interview topic and fieldnote guides

Appendix 1: Recruitment and consent processes for in-depth interviews and participant observations

Recruitment and consent processes for in-depth interviews

**Hostel staff & managers**

Hostel managers initially approached by researcher and if they agreed researcher met with them to explain the study.

**Older people with memory problems**

Researchers met with managers and staff to identify potentially eligible hostel residents, deemed by staff to have capacity* to consent to participate.

**Health and social care practitioners**

Potential health and social care practitioners were emailed with an information sheet and invited to take part in an interview.

Researcher attended team meetings to introduce the study and invite staff to participate in an interview, interested staff were given a participant information sheet.

Individual face to face (hostel staff and residents) or online (health and social care practitioners) meetings were arranged with potential participants to discuss the study, answer questions and take informed consent.

Hostel staff approached older people with memory problems, explained the study, provided an information sheet and sought their agreement for a researcher to approach.

*Researcher assessed capacity to make this decision at the point of consent. All participants gave written or audio-recorded informed consent. We did not recruit older people who lacked capacity to consent.

* Mental capacity to give informed consent was initially judged by experienced hostel staff who knew the participants best. This was following discussion and guidance by the team based on the Mental Capacity Act (2005) (England and Wales). This guidance was followed by trained researchers undertaking a brief assessment of the potential participant’s capacity to give informed consent as part of the consenting procedure.

Recruitment and consent processes for participant observations

**Hostel managers**

Hostel managers who participated in interviews initially approached by researcher to explain the study. If willing to participate researcher will take consent from manager for hostel participation.

**Older people with memory problems**

Researcher met with managers and staff to identify potentially eligible hostel residents, deemed by staff to have capacity to consent to participate.

**Hostel staff**

Researcher attended team meetings to introduce the study and invite staff to participate, interested staff were given a participant information sheet.

Individual face to face meetings were arranged with potential participants to discuss the study, answer questions and take informed consent.

Hostel staff approached older people with memory problems, explained the study, provided an information sheet and sought their agreement for a researcher to approach.

Researcher assessed capacity to make this decision at the point of consent. All participants gave written or audio-recorded informed consent. We did not recruit older people who lacked capacity to consent.

**Non-participating hostel residents or visitors**

**Are they directly interacting with participants with memory problems?**

**Non-participating staff present in team meetings being observed**

At first meeting researcher will introduce self and provide information sheet with information on how to opt-out. Staff invited to email researcher if wishing their contribution to be included in field notes. In later meetings researcher announces themselves as observer.

Individual consent not sought – observations are ‘general’ and do not include any identifiable information and restricted to general atmosphere or scene. If distressed or uncomfortable researcher will leave.

**No**

**Yes**

Staff or researcher will approach and invite participation and give participant information sheet and answer questions and if they agree take written informed consent.

**Before**

**beginning**

**observations**

**During**

**observations**

Principles informing capacity assessments by staff and research team based on Mental Capacity Act (2005)
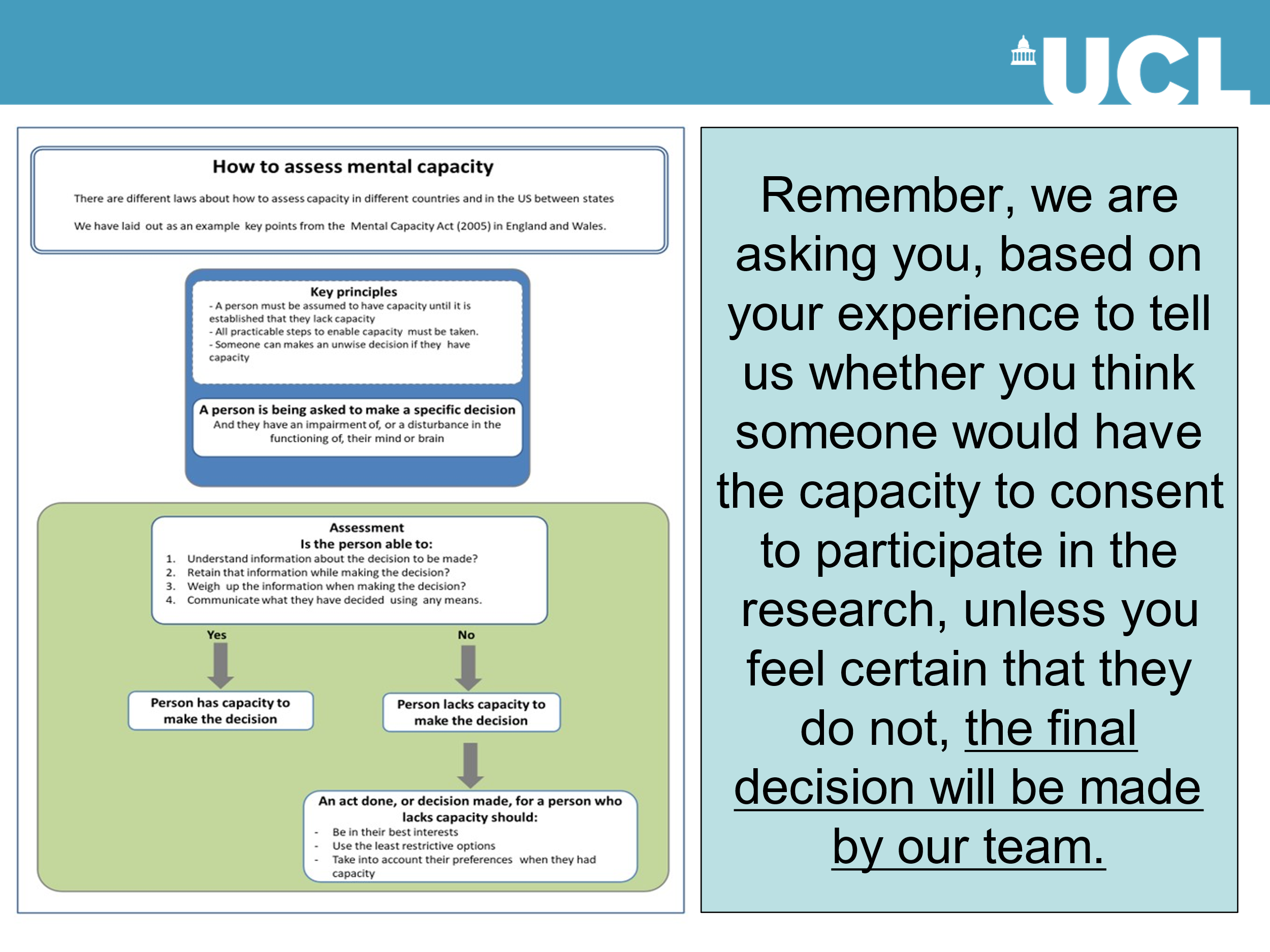


Appendix 2: Interview and fieldnote topic guides

**Interview topic guide for hostel staff and managers**

Thank you for agreeing to talk with me. This part of our study is to help us understand better how to support people who are experiencing homelessness and living with memory problems.

In order to make sure that I don’t miss anything, I will record our conversation on a digital recorder and then it will be professionally transcribed. Whilst we are talking try not to use anyone’s names or information which may identify them. Once the interview has been transcribed, I will ensure that all identifying characteristics are removed so that you, your organisation and anyone you talk about cannot be identified. Once again, everything you tell me will be treated with complete confidence unless you indicate that you or someone else may be at risk of serious harm. If at any point during the interview you feel that you need to stop or leave the room please do tell me. Do you have any questions?

**Interview**

**To start, could you tell me a little about your work? How are you involved with caring for people experiencing homelessness and or memory problems?**

- Can you describe (without using name) a particular older person who you have worked with and what their difficulties were?
- How did you make sense of and understand their difficulties?
- How did supporting them differ from caring for residents without memory problems?
- How did their memory difficulties impact upon their day-to-day life and wellbeing?
- How did their memory difficulties get in their way of moving to or being in settled accommodation?
- Who else (if anyone) was involved in their care and support from outside the hostel team?

**What do you see as the main challenges in supporting older people with memory problems who are experiencing homelessness and what might help?**

(Prompt in terms of accessing support from outside, offering care and support within the hostel, supporting people to move on from the hostel)

- Challenges / solutions at a service level?
- Challenges / solutions at a team level?
- Challenges / solutions at an individual staff level?
- Challenges related to the individual with memory problems?

**What do you think ‘best practice’ would look like in supporting older people with memory problems who are experiencing homelessness in your hostel?**

- What would good health care look like? (memory assessment, primary care)
- What would good social care look like? (personal care needs, accessing in hostel home care)
- What would help people to move on from homelessness / hostel / temporary accommodation? (where do people with memory problems typically move on to)

**What do you think would be important positive outcomes for older people with memory problems who are experiencing homelessness?**

- What might this look like in practice?
- What / who might help them to achieve these outcomes?
- What factors might get in the way of these positive outcomes?

**We want to develop a support intervention for hostel staff supporting older residents with memory problems. What do you think it would be important to include?**

- What areas or topics would it be important to address? (e.g. recognising memory problems, referral pathways, communication, understanding challenging behaviours, managing risky behaviours)
- Is there any specific training that would be useful for you/your team?
- What may help/make it harder for you/your team to put support strategies and new learning into practice?
- In addition to training what additional support would be useful for you/your team?

**What knowledge and skills might frontline staff in homelessness organisations need to support older people experiencing memory problems?**

- What could help them to build these skills?
- What have you learnt from approaches in other related areas (e.g. dementia care, support in homelessness, drug and alcohol support etc)

**Thank you - Is there anything else you would like to add?**

**Interview topic guide for people with memory problems**

Thank you for agreeing to talk with me. This part of our study is to help us understand better how to support people who are experiencing homelessness and living with memory problems.

To make sure that I don’t miss anything, I will record our conversation on a digital recorder and then it will be typed up. Whilst we are talking try not to use anyone’s names. I will take out any named or places after it is typed to make sure you cannot be identified. Once again, everything you tell me will be treated with complete confidence unless you indicate that you or someone else may be at risk of serious harm.

If at any point during the interview you feel that you need to stop or leave the room please do tell me. Do you have any questions?

**Interview**

**To start, could you tell me a little about yourself? Have you noticed any changes in your memory as you have got older?**

- What changes have you noticed?
- How does it affect what you can do (prompt re functioning, taking care of self, managing money, cooking, cleaning, seeing people, washing).
- What do you think may have caused these problems or may make them worse?
- Have you had any help or support with your memory problems?

**How do you like to spend your time during the day?**

- What is important to you?
- What are your hopes for the future?
- What makes it harder for you to do what you would like?

**How do your memory problems get in the way of doing what you would like?**

- Did / do your memory problems make it harder to move on from a hostel?

**While living in a hostel/ experiencing homelessness what support was/is most helpful?**

(Prompt in terms of accessing support from outside, offering care and support within the hostel, supporting people to move on from the hostel)

- Did you need any extra or special help because of your memory problems, and what was this?
- What helped you to move on (if relevant)

**While living in a hostel/ experiencing homelessness what support was/is not helpful?**

- Was anything missing?
- What didn’t you like?

**We want to develop a support intervention to help hostel staff supporting older residents with memory problems. What do you think it would be important to include?**

- What would you tell hostel staff about what is important to you?
- What advice would you give them?

**Thank you - Is there anything else you would like to add?**

**Interview topic guide for health and social care staff**

Thank you for agreeing to talk with me. This part of our study is to help us understand better how to support people who are experiencing homelessness and living with memory problems.

In order to make sure that I don’t miss anything, I will record our conversation on a digital recorder and then it will be professionally transcribed. Whilst we are talking try not to use anyone’s names or information which may identify them. Once the interview has been transcribed, I will ensure that all identifying characteristics are removed so that you, your organisation and anyone you talk about cannot be identified. Once again, everything you tell me will be treated with complete confidence unless you indicate that you or someone else may be at risk of serious harm. If at any point during the interview you feel that you need to stop or leave the room please do tell me. Do you have any questions?

**Interview**

**To start, could you tell me a little about your work? How are you involved with caring for people experiencing homelessness and or memory problems?**

- Can you describe (without using name) a particular older person who you have worked with and what their difficulties were?
- How did you make sense of and understand their difficulties?
- How did their memory difficulties impact upon their day to day life and wellbeing?
- How did their memory difficulties get in their way of moving to or being in settled accommodation?

**What do you see as the main challenges in supporting older people with memory problems who are experiencing homelessness and what might help?**

- Challenges at a service level?
- Challenges at a team level?
- Challenges at an individual staff level?
- Challenges related to the individual with memory problems?

**What do you think ‘best practice’ would look like in supporting older people with memory problems who are experiencing homelessness?**

- What would good health care look like?
- What would good social care look like?
- What would help people to move on from homelessness / hostel / temporary accommodation?

**What do you think would be important positive outcomes for older people with memory problems who are experiencing homelessness?**

- What might this look like in practice?
- What / who might help them to achieve these outcomes?
- What factors might get in the way of these positive outcomes?

**We want to develop a support intervention for hostel staff supporting older residents with memory problems. What do you think it would be important to include?**

- What areas or topics would it be important to address?
- Is there any specific training that would be useful?
- What may help/make it harder for frontline staff to put support strategies and new learning into practice?

**What knowledge and skills might frontline staff in homelessness organisations need to support older people experiencing memory problems?**

- What could help them to build these skills?
- What could we learn from approaches in other related areas (e.g. dementia care, support in homelessness, drug and alcohol support etc)

**Thank you - Is there anything else you would like to add?**

**Title: Older, homeless and experiencing memory problems: An observational study**

Keep two separate note books - one for recording both short notes and more detailed field notes and the other for keeping a reflective diary. To maintain confidentiality of participants please do not record any identifiable information in your field notes. Use ID numbers, or if easier create pseudonyms for any participants.

Please follow processes outlined in the protocol (Version x) in terms of who is referred to in field notes. Distinguish between “intensive” and “general” observations with intensive observation attributable to individuals who have consented to participate in the study and general observation attributable to no one in particular, but focused on overall atmosphere of a scene or setting.

**Guide for completing field notes**

**Visit details: Observations of staff meeting/discussions (*complete a new sheet for each observation*)**

**Type of observation (e.g. training session, team meeting, staff handover) …………………………..............................................................................................**

**Who was present (list study IDs if participants and/or job titles): …………………………......................................................................................................................................................................................................................**

**Observation visit date: ……………………………………………..**

**Observation visit start time: ……………………………………..**

**Observation visit end time: ……………………………………….**

**Researcher observing: ……………………………………………….**

**Guidance: Areas of interest to keep in mind during the observation of meetings:**

1. **A practical overview purpose of meeting**
2. Within an observation, record the length of times of discussion pertaining to participating residents in field notes.
3. note who is present (follow procedures for those who have and have not participated outlined in protocol)
4. the environment, including where the meeting takes place e.g. private office
5. “atmosphere” including general feelings about tension, is it welcoming, comfortable etc
6. Any specific care and support tasks that are discussed in relation to residents with memory problems
7. **Discussions related to the care and support of participating residents**
8. how person with memory problems (including behaviour, language, ethnicity and culture), staff, team and/or management and organisational factors impact on discussion of support provided
9. the language and communication used to discuss participating residents
10. whether additional unmet needs are discussed by staff and plans for managing and addressing these
11. how your presence as a participant observer may have influenced your observations

**Field notes *(Type up field notes here)***

**Hostel visit details (*complete a new sheet for each visit/observation*) and field notes guide for staff/client observations**

**Staff ID (any participating staff during observation):**

**Client ID (any participating staff during observation):**

**Observation visit date: …………………………………………….**

**Observation visit start time: …………………………………….**

**Observation visit end time: ……………………………………..**

**Researcher observing: ……………………………………………..**

**Guidance: Areas of interest to keep in mind during the observation:**

1. **A practical overview of the visit**
2. Within an observation, record the length of times of staff/resident interactions in field notes.
3. note who is present (follow procedures for those who have and have not participated outlined in protocol)
4. the environment, including physical layout, decor and cleanliness
5. “atmosphere” including general feelings about tension, is it welcoming, comfortable etc
6. Any specific care and support tasks that are delivered
7. **Interactions and responses of participating staff with participating residents and others**
   - *positive, negative and neutral interactions/responses*
   - *support wellbeing/choice/autonomy/engagement where possible*
   - *challenges to wellbeing (e.g. symptoms of distress, refusal of care or risks) and responses to it where this occurs, and whether these strategies are effective in resolving distress and enabling necessary care and support to be given*
   - *emotional responses, strategies and resources used (e.g. practical, social, spiritual)*
   - *Language and communication used and how this impacts on interactions*
8. how person with memory problems (including behaviour, language, ethnicity and culture), staff, team and/or management and organisational factors impact on support provided
9. whether additional unmet needs arise, either stated by the person with memory problems or staff and how these are managed.
10. general thoughts and feelings about the care and support observed and how this promotes wellbeing.
11. how your presence as a participant observer may have influenced your observations

**Field notes** (***Type up field notes here)***
